# Radiation Levels from Toilets Used By Patients Injected with 99mTc-based Radiopharmaceuticals in Ibadan

**DOI:** 10.4274/mirt.82905

**Published:** 2014-06-05

**Authors:** John Enyi Ejeh, Tolulope Hadrat Abiodun, Kayode Solomon Adedapo, Yetunde Ajoke Onimode, Olusegun Akinwale Ayeni

**Affiliations:** 1 Ibadan University College Hospital, Department of Nuclear Medicine, Ibadan, Nigeria; 2 Ibadan University College of Medicine, Department of Chemical Pathology, Ibadan, Nigeria; 3 Ibadan University College of Medicine, Department of Radiotherapy, Ibadan, Nigeria

**Keywords:** Technetium 99m, radiopharmaceuticals, nuclear medicine, radiation monitoring, Radiation protection

## Abstract

Objective: The use of radionuclides in patients undergoing nuclear medicine procedures presents a special concern on the safety of not only the patients but also of those who come in contact with such patients either at the nuclear medicine centre or at home after discharge from the facility. This has heightened the public concern about nuclear medicine especially in Nigeria where the practice is new. When patients are injected with radioactivity for nuclear medicine procedures they excrete most of the radioactivity via urine even before leaving the nuclear medicine facility. Therefore, we set out to survey the toilets used by these patients in the hospital for radiation levels to know the radiation risk posed by the ‘radioactive urine’ of the patients to the general public and radiation workers respectively.

Methods: A portable digital radiation survey meter was used for measurement of radioactivity in toilets used by a total number of 202 patients injected with 99mTc-based radiopharmaceuticals over a period of 60 days for the level of radioactivity.

Results: The minimum background radiation level measured was 0.18 µSv/h while the maximum was 0.44 µSv/h and the mean background reading was 0.28 µSv/h. The readings recorded for the male toilets were: 0.1 µSv/h minimum, 5.62 µSv/h maximum with a mean of 0.52 µSv/h while those for the female patients were 0.19 µSv/h minimum, 21.73 µSv/h maximum and a mean of 3.3 µSv/h.

Conclusions: In conclusion, the radiation levels from toilets used by patients injected with 99mTc-based radiopharmaceuticals were within reasonable and acceptable limits and do not pose significant radiation risk to others.

## Abstract (tr)

**Amaç:** Nükleer Tıp uygulamaları yapılan hastalarda radyonüklidlerin kullanılması, yalnızca hastalarda değil, aynı zamanda bu hastalarla nükleer tıp merkezinde veya fasiliteden ayrıldıktan sonra evde temas eden kişilerde de güvenlikle ilgili kaygılar uyandırmaktadır. Bu durum özellikle uygulamanın yeni olduğu Nijerya’da Nükleer Tıp ile ilgili halkta bir kaygı yaratmıştır. Nükleer Tıp uygulamaları için hastalara radyoaktivite enjekte edildikten sonra, hastalar radyoaktivitenin çoğunu hatta fasiliteden ayrılmadan önce idrar yoluyla atmaktadır. Bu nedenle, hastanede hastaların kullandıkları tuvaletlerde radyasyon düzeylerini inceleyerek, hastaların radyoaktif idrarlarının genel halk ve radyasyon çalışanlarına yarattığı radyasyon riskini araştırdık.

**Yöntem:** Tc 99m işaretli radyofarmasötik enjekte edilmiş olan toplam 202 hasta tarafından kullanılan tuvaletlerdeki radyoaktivite düzeyi, 60 gün süreyle, taşınabilir bir dijital radyasyon ölçüm cihazı kullanılarak ölçüldü.

**Bulgular:** Ölçülen minimum zemin aktivite değeri 0,18 µSv/saat, maksimim değer 0,44 µSv/saat ve ortalama zemin değeri 0,28 µSv/saat idi. Erkek tuvaletlerinde yapılan ölçümler minimum 0,1 µSv/saat, maksimum 5,62 µSv/saat, ortalama 0,52 µSv/saat iken kadın tuvaletlerinde bu değerler minimum 0,19 µSv/saat, maksimum 21,73 µSv/saat ve oratama 3,3 µSv/saat idi.

**Sonuç:** Sonuç olarak, Tc 99m işaretli radyofarmasötik enjekte edilen hastaların kullandığı tuvaletlerdeki radyasyon düzeyleri makul ve kabul edilebilir düzeylerde bulunmuştur ve diğer kişilere önemli bir radyasyon riski oluşturmamaktadır.

## INTRODUCTION

The practice of nuclear medicine imaging involves administering a compound, which is labeled with a gamma ray emitting or positron emitting radionuclide into the body of patients resulting in gamma ray emissions from the patients until their activity becomes negligible. These are used to provide diagnostic information in a wide range of disease states and they range from those with short half lives such as 15O (T1/2=123 s) emitting photons of 511 keV to relatively long lived ones such as 131I (T1/2=8.04 d) with gamma ray energies of 636.9 keV (7.3%) and 364.5 keV (81.2%) and beta minus (β-) energy of 606.3 keV (89.3%) (1). Some of these radionuclides are also used for therapeutic purposes. Technetium-99m (^99m^Tc) with gamma ray energy of 140 keV and a half life of 6.02 h is today the most widely used radionuclide in nuclear medicine (2,3). When injected into patients, ^99m^Tc is excreted mostly via the renal pathway primarily by glomerular filtration. It is said that in patients with normal renal function, 50% to 60% of the injected dose is excreted in the urine within 24 h (3). 

The use of radionuclides in patients undergoing nuclear medicine procedures presents special concerns for evaluation of radiation dose and risk to the population (4) and the general public (5) including relatives, friends and others who come in contact with the patients. 

Based on the need to protect the general public from radiation exposure from the environment including that from patients administered with radionuclides, the International Commission on Radiological Protection (ICRP) and the Atomic Energy Regulatory Board (AERB) of India set the acceptable annual exposure dose for the general public to be 1mSv, while the European commission linked the constraint to age (children, including the unborn child - 1 mSv, adults up to 60 years old - 3 mSv, adults more than 60 years old - 15 mSv and general public - 0.3 mSv) (5). The International Atomic Energy Agency (I.A.E.A) set the limit at 5 mSv (6) and the Nuclear Regulatory Commission (NRC) of the US set a limit of less than 5 mSv (7). 

Nuclear medicine is relatively new in Nigeria with the first nuclear medicine department established in 2006 at the University College Hospital Ibadan. The phobia for radiation which has led to a heightened public concern of medical procedures involving radiation (8) is still very high in the country. The major concern is the fate of those who come in contact with these patients after they have been discharged from the nuclear medicine facility. Previous work had shown that patients administered radionuclides for nuclear medicine procedures and who are emitting radiation do so at levels that are far below the limits set by radiation protection regulations (9,10) and hence does not pose any significant radiation risk to the environment. It is also on record, according to the National Council on Radiation Protection and Measurement (NCRP), that in the US population the contribution of nuclear medicine to the total average effective dose equivalent, which is 360mrem/yr, is a paltry 4% (14 mrem/yr or 0.14 mSv/yr) compared to medical x-rays which contribute 11% (39 mrem/yr or 0.39 mSv/yr) (11,12) 

The nuclear medicine patient, before leaving the nuclear medicine facility, would have eliminated much of the radiation by both physical and biological decay of the radionuclide. The patients are usually encouraged to drink large amounts of water to aid the excretion of the radionuclide injected from the body and for the attenuation of the radiation dose to the bladder (13). Since the patients excrete much of the radiation via urination in the nuclear medicine facility before discharge, we decided to investigate the radiation levels in toilets used by these patients after injection of ^99m^Tc-based radiopharmaceuticals. 

This study was therefore aimed at investigating the radiation levels in the toilets to know the radiation risk posed by the “radioactive urine” to relative of patients (who accompany very sick patients to such toilets), the cleaners and other radiation personnel who come in contact with such toilets in the course of their work. This will enable us review the radiation safety rules in the toilets used by patients undergoing scintigraphy using ^99m^Tc-based radiopharmaceuticals. 

## MATERIALS AND METHODS

A portable digital dose rate meter (Radiagem 2000- Canberra Eurisys, Montigny-le-Bretonneux) with a measuring range of 0.1 µSv/h to 100 µSv/h (energy range of 40 keV to 1.5 MeV) and a sensitivity of 0.83 c/s per µSv/h was used to measure the gamma dose rates in the toilets over a period of 60 days. Background readings for gamma dose rate in the toilets were taken, usually between 7.30 - 8.00 am prior to injection of radiopharmaceutical and use of the toilets by the patients for each day during this period. Measurements were also taken at 9.00 am shortly after the patients injected would start using the toilets. Further measurements were carried out at 12.00 pm and 3.00 pm respectively for each day, during which time the use of the toilet would have reached its peak. Toilets used were the normal WC found in most homes in our country and measurements were taken directly above the toilet seat, at the sides of the seat and at 1m from the toilet seat. These were carried out both in the male and female toilets. 

## RESULTS

A total of 202 patients injected with 99m^Tc^-based radiopharmaceuticals used the toilets during the period of survey. We had 60 male patients and 142 female patients respectively. The summary of the demographics and amount of radionuclide injected into the patients is shown in Table 1. 

A total of 1,745 measurements were carried out within the period of survey of the toilets. Table 2 shows the summary of total number of measurements carried out while Table 3 shows a summary of results of the measurements carried out. Table 3 also shows that the dose rate values in the female toilet were much higher than those of their male counterpart and the highest dose rate values were 1 order of magnitude higher than dose limits for restricted areas (≤ 10 µSv/h). The mean dose rate recorded for male toilets was also lower than the mean for the female toilets. 

## DISCUSSION

Worldwide, radiation protection is a very important aspect of nuclear medicine practice (14,15,16,17,18,19,20,21). Measures put in place to ensure radiation safety is first from the “ALARA” (As Low As Reasonably Achievable) principle, to giving of specific instructions to the radiation personnel, the patients and their relations and care givers. These instructions also include radiation caution signs and warnings, making up a comprehensive and remarkably consistent framework for radiation protection of patients undergoing Nuclear medicine procedures. All these are aimed at containing radioactivity emitted or excreted by patients that have undergone nuclear medicine procedures. 

The radioactivity is also contained by the use of toilet facility either at the nuclear medicine centre or at the patients’ home. Usually, the majority of radioactive materials used in nuclear medicine have short half lives. This means that the radionuclides decay to background radiation in a short while. Technetium-99m for instance, has a half life of 6.02 h and this means that in one day (24 h) it would have decayed physically by a factor of 4. So, the period during which the patients stay in the hospital before discharge is enough for much of the injected radioactivity to decay to safe levels. 

On a general note, radioactivity deposited internally in nuclear medicine patients is not a likely source of significant dose and risk for staff and relatives as it is deemed to be low as long as the patient is not incontinent or prone to vomit (22,23). Radiation levels in unrestricted areas should deliver a radiation dose of less than 0.5 µSv/h (0.05 mrem/h) assuming continuous occupation of the area. Transient radiation levels of up to 20 µSv/h (2 mrem/h) are permitted, provided that the 1 mSv/yr (0.1 rem/yr) limit for individual members of the public is not exceeded (2). 

From the results of our measurements, we observed that the maximum radiation level recorded was 21.73 µSv/h which in comparison with the above limits, is seen to be within acceptable limits. The highest dose rate measured in the male toilet was 5.62 µSv/h which is far below the limits set for restricted areas, such as the radioactive based toilets. Data obtained from this study also revealed that when the toilets are not flushed, the radiation levels could be two orders of magnitude higher than the dose limits for controlled areas but are less than the dose limits when the toilets are flushed twice after use. The high levels recorded in the female toilets could be attributed to the fact that during the period of survey, the female toilet was more frequently used than the male toilets, since the male patients with catheter did not use the toilets whereas there was no female patient with catheter. It could also be attributed the fact that sometimes, instructions to flush the toilet twice after use were not followed. 

At the nuclear medicine centre where substantial radioactivity is meant to be excreted, is also the regulation of the need for a delay tank. Furthermore, the issue of delay tank ensures that the public is not exposed to any radiation hazard as the waste is only discharged when the radioactivity would have reached background levels.

## CONCLUSION

We can therefore conclude that the radiation levels from toilets used by patients injected with ^99m^Tc-labeled radiopharmaceuticals were within reasonable and acceptable limits and do not pose significant radiation risk to others. However, these radiation levels can further be significantly reduced if radiation safety instructions are properly followed by the patients. Secondly, usage of automatic toilet flushers, which will flush the toilets should the patient fail to follow the instruction to flush twice after use will be of great efficacy especially within the milieu of high illiteracy. 

It could also be inferred from this study that, when patients are discharged home from nuclear medicine facilities after been injected with ^99m^Tc-based radiopharmaceuticals, they would pose no radiation danger to their family members. 

## ACKNOWLEDGEMENTS

The authors are grateful to Prof. Mboyo D. T. Vangu of University of Witwatersrand, Johannesburg South Africa for suggesting that we should carry out this work.

## Figures and Tables

**Table 1:**
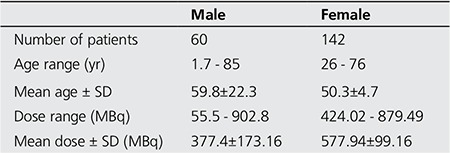
Patient Demographics and amount of radioactivity injected

**Table 2:**
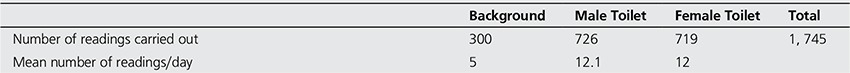
Number of measurements carried out

**Table 3:**
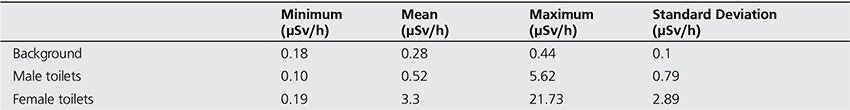
Dose Rate from toilets surveyed
